# Hand hygiene excellence award winner: spreading the experience countrywide in Romania

**DOI:** 10.1186/2047-2994-4-S1-P148

**Published:** 2015-06-16

**Authors:** A Moldovan, M Capilna, I Cotoara, B Moldovan

**Affiliations:** 1 Prevention and Control of Infections, Sf Constantin Hospital, Brasov, Romania; 2 Obstetrics& Gynecology, Sf Constantin Hospital, Brasov, Romania; 3 General Surgery, Sf Constantin Hospital, Brasov, Romania

## Background

Hospital « Sf.Constantin » was designated among the winners of the « European Hand Hygiene Excellence Award » ( 2013). This award turned the winner medical team into a promoter of infection control, as part of the missions given to HHEA awardees.

## Objectives

Improving hygiene in Romania.

## Methods

The promotion of actions was conducted as: know-how in hospitals; organization of workshops/courses (infectious diseases, epidemiology, use of invasive devices, prevention of bloodstream infections, antibiotic stewardship); co-organization of two annual workshops with international participation; initiating the 5 May (WHO hand hygiene day) celebrations in Romania; translation into Romanian of books in the field.

## Results

Since 2013, we conducted 29 courses/workshops in 16 towns. The number of participants was 1530. The topics encompassed 17 themes. All presentations were made in the spirit of the economy of peace, i.e. pro-bono. We gave the materials, initiated on-line feed-back. We contributed to the completion of four master theses, and we are are doing the initiation of a patient’s education program. Instructed professionals came from surgery (51%), oncology (27%), intensive care (16%) and medicine (6%), being registered nurses (65%), doctors (25%), other (10%). We are involved in three major projects in regards to the implementation of infection control measures.

## Conclusion

As HHEA winner, our institution contributes to spread infection control/hospital epidemiology in Romania. Our experience verifies the idea of considering hand hygiene as the entrance door to infection control.

## Disclosure of interest

None declared.

